# Comparison of the three-dimensional organization of sperm and fibroblast genomes using the Hi-C approach

**DOI:** 10.1186/s13059-015-0642-0

**Published:** 2015-04-14

**Authors:** Nariman Battulin, Veniamin S Fishman, Alexander M Mazur, Mikhail Pomaznoy, Anna A Khabarova, Dmitry A Afonnikov, Egor B Prokhortchouk, Oleg L Serov

**Affiliations:** Institute of Cytology and Genetics, Novosibirsk, 630090 Russia; Novosibirsk State University, Novosibirsk, 630090 Russia; Center ‘Bioengineering’, Russian Academy of Sciences, Moscow, 117312 Russia; National Research Center, Kurchatov Institute, Moscow, 123098 Russia; Skoltech Center for Stem Cell Research, Skolkovo Institute of Science and Technology, Skolkovo 143025, Moscow, Russia

## Abstract

**Background:**

The three-dimensional organization of the genome is tightly connected to its biological function. The Hi-C approach was recently introduced as a method that can be used to identify higher-order chromatin interactions genome-wide. The aim of this study was to determine genome-wide chromatin interaction frequencies using the Hi-C approach in mouse sperm cells and embryonic fibroblasts.

**Results:**

The obtained data demonstrate that the three-dimensional genome organizations of sperm and fibroblast cells show a high degree of similarity both with each other and with the previously described mouse embryonic stem cells. Both A- and B-compartments and topologically associated domains are present in spermatozoa and fibroblasts. Nevertheless, sperm cells and fibroblasts exhibit statistically significant differences between each other in the contact probabilities of defined loci. Tight packaging of the sperm genome results in an enrichment of long-range contacts compared with the fibroblasts. However, only 30% of the differences in the number of contacts are based on differences in the densities of their genome packages; the main source of the differences is the gain or loss of contacts that are specific for defined genome regions. We find that the dependence of the contact probability on genomic distance for sperm is close to the dependence predicted for the fractal globular folding of chromatin.

**Conclusions:**

Overall, we can conclude that the three-dimensional structure of the genome is passed through generations without being dramatically changed in sperm cells.

**Electronic supplementary material:**

The online version of this article (doi:10.1186/s13059-015-0642-0) contains supplementary material, which is available to authorized users.

## Background

For a long time, the study of chromosome architectures was based on fluorescence-based microscopy [1-3]. The approach allowed researchers to establish that individual chromosomes are localized in distinct spaces designated as chromosome territories [4]. Moreover, chromosome territories in nuclei are localized in a non-random manner with respect to the nuclear periphery [4] and are able to interact and form gene clusters that loop out of their chromosome territory [5]. The development of a technique based on chromosome conformation capture (3C) [6] and related methods (4C, 5C and Hi-C) [7-10] significantly extended the possibility of studying the three-dimensional genome architecture. The Hi-C technology, as a genome-wide approach, allows the determination of the contact frequency between any pair of loci within 10 to 100 nm at the moment of nuclei fixation [11]. Thus, Hi-C provides ‘a true all-by-all genome-wide interaction map’ [11] based on the quantitative estimation of proximity-ligation events for millions of loci in the genome. Importantly, the Hi-C interaction frequencies are well correlated with the mean spatial distance separating loci, as measured using independent methods such as FISH [12,13], indicating that the Hi-C data can accurately reproduce the expected distance.

Genome-wide Hi-C mapping has revealed that inter- and intrachromosomal interactions are represented by two compartments, A and B, which have a mean size of approximately 5 Mb each [10,14,15]. Loci of the A compartments interact preferentially with loci of other A compartments, while the B compartments often are in contact with other B compartments. Additionally, genome-wide Hi-C mapping, in combination with a hidden Markov model, revealed that human and mouse chromosomes are composed of approximately 2,200 topologically associated domains (TADs) that have a median size of 880 kb and cover over 90% of the genome [16]. The same conclusion was simultaneously made based on the 5C analysis of the mouse X-chromosome inactivation center [17]. It is important to note that the topological domains are stable across different cells (mouse embryonic stem (ES) cells and mouse cortex or human ES cells and human IMR90 fibroblasts) and highly conserved across species (human and mouse), ‘indicating that topological domains are an inherent property of the mammalian genome’ [16].

In mammals, chromatin organization in mature sperm cells is unique among cell types. The genome of sperm cells is packaged in a highly condensed configuration. This packaging enables more than a 10-fold decrease in nucleus size in spermatozoa relative to the somatic interphase nucleus. This extraordinary compactness results from the replacement of histones with protamines. Protamines coil sperm DNA into toroids that form an almost crystalline structure. Only 1 to 15% of mammalian sperm DNA is bound to histones rather than protamines [18]. Additionally, sperm cells have a haploid, transcriptionally inactive set of chromosomes [18,19]. It is unknown how all of the aforementioned features affect the three-dimensional organization of the sperm genome.

The aim of this study is to compare the three-dimensional genome architectures of sperm cells and fibroblasts, as somatic cells, using the Hi-C approach. The obtained results demonstrate that genome-wide interaction maps of mouse sperm and fibroblast genomes show a high degree of similarity both to each other and to the previously described Hi-C organization of mouse ES cells. Nevertheless, there are statistically significant differences in the spatial contacts of some regions.

## Results

We created Hi-C libraries from mouse fibroblasts and mature sperm cells using the tethered conformation capture (TCC) protocol developed by Kalhor and colleagues [13]. The TCC method allows one to significantly reduce the noise obtained using the Hi-C approach, particularly the noise from interchromosomal interactions. We performed massive parallel sequencing of the Hi-C libraries at a depth of 150 and 400 million read pairs for fibroblasts and sperm, respectively, and filtered the data so that the reads could be uniquely aligned to the mouse genome reference sequence.

**Figure 1:**
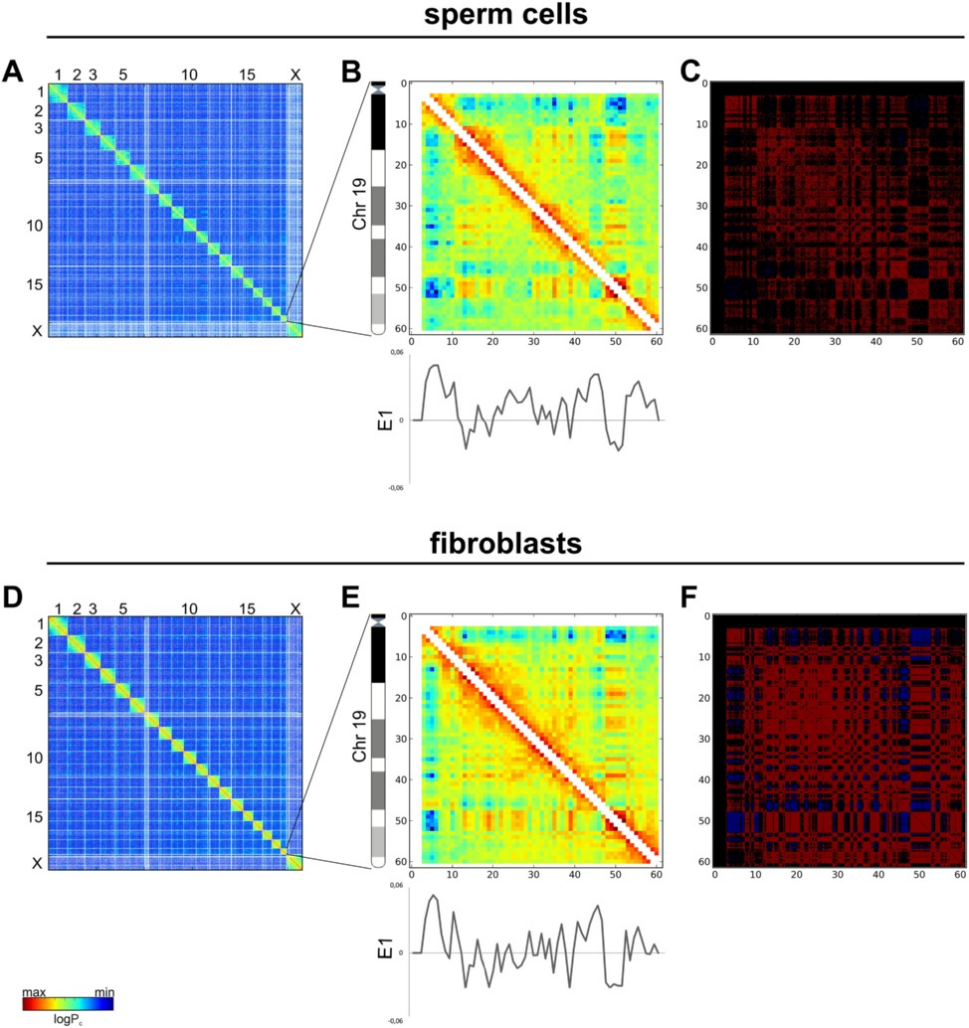
Relative Hi-C contact probability maps. Whole-genome **(A,D)** and chromosome 19 **(B,E)** maps at a 1 Mb resolution for sperm cells and fibroblasts. The color of each dot represents the log of the interaction probability for the corresponding genome bins. The graphs under the heatmaps show E1 values for chromosome 19 in sperm cells and fibroblasts. The two-dimensional contact correlation matrices constructed as in [10] **(C,F)** demonstrate the characteristic plaid patterns for both sperm cells and fibroblasts.

Figure 1 presents genome-wide and chromosome 19 Hi-C maps for sperm cells and fibroblasts (binned at 1 Mb resolution) as heatmaps, where the color indicates the contact frequency. Both interaction maps display visibly similar plaid patterns of the regional enrichment or depletion of long-range interactions. Individual chromosomes visually rise above both heatmaps due to the enrichment of contacts.

**Figure 2:**
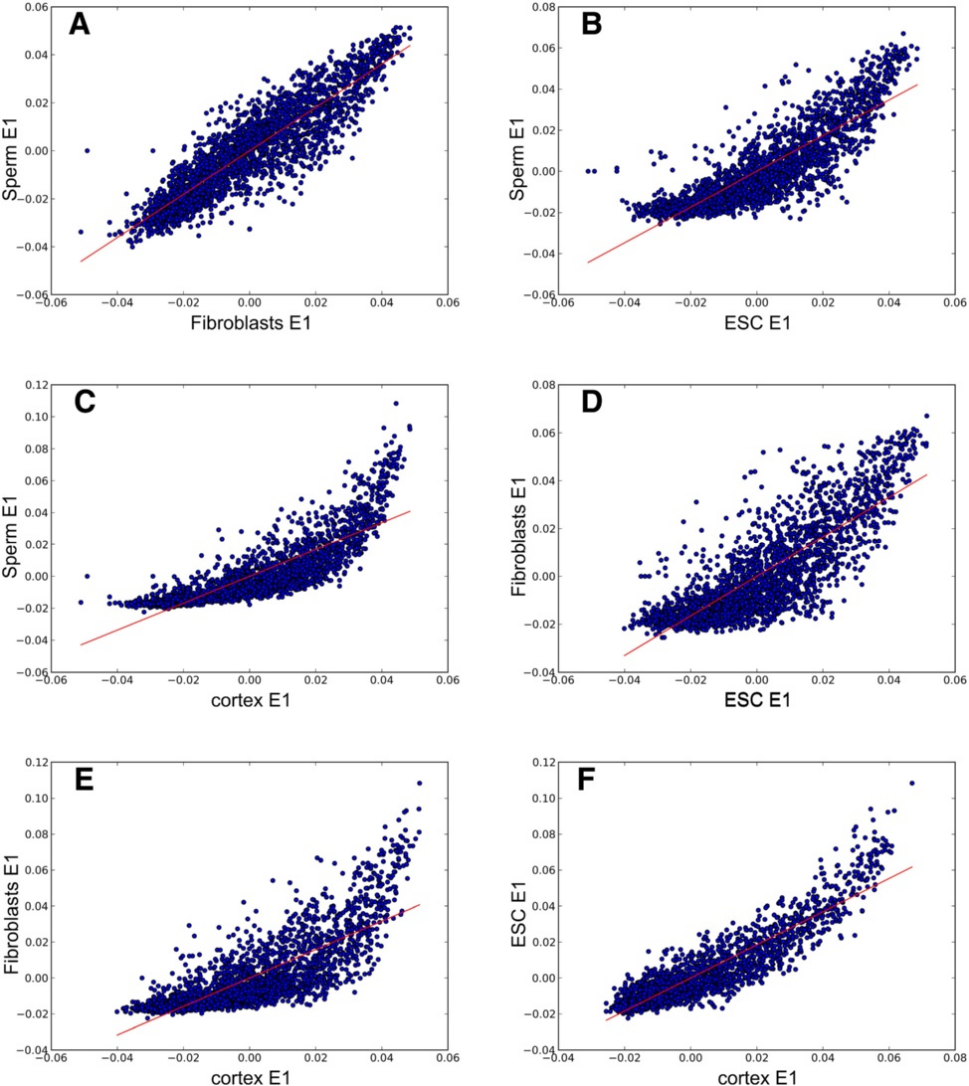
Comparison of the E1 values for sperms cells, fibroblasts, cortex and ES cells. **(A-F)** Scatter plots of eigenvectors. The E1 values are highly similar in fibroblasts and sperm cells. The x- and y-axes indicate the E1 values from sperm and fibroblasts **(A)**; sperm and ES cells (ESC) **(B)**; sperm and cortex **(C)**; fibroblasts and ES cells **(D)**; fibroblasts and cortex **(E)**; ES cells and cortex **(F)**. The line represents the linear trend for the obtained values.

A previous study showed that contact heatmaps could be decomposed into a set of eigenvector tracks. The first eigenvector (E1) values correlate with different genome properties such as replication time, GC content and histone marks [15]. We performed eigenvector decomposition and compared the obtained E1 values for spermatozoa, fibroblasts, ES cells and cortex using the Hi-C data published by Dixon and colleagues [16] (Figures 1 and 2). Again, one can note a high degree of similarity between spermatozoa and fibroblasts (Spearman r = 0.899), as well as between spermatozoa and ES cells (Spearman r = 0.878) and between spermatozoa and cortex (Spearman r = 0.901) (Additional file 1). Similar results were obtained when another measure of dependence for two-variable relationships, maximal information coefficient [20], was used to measure relationships between E1 values for different datasets (Additional file 1). Lieberman-Aiden *et al*. [10] suggested that the genome could be divided into discrete A and B compartments that are characterized by positive and negative E1 values. Further extension of this work propose a continuous set of domains that are characterized by similar E1 values for regions inside a compartment [15]. Other research also supported this viewpoint [11]. Thus, the similarities of E1 values imply a similar distribution of A and B compartments in sperm cells and fibroblasts, emphasizing conservation of genome organization (Figures 1 and 2).

**Figure 3:**
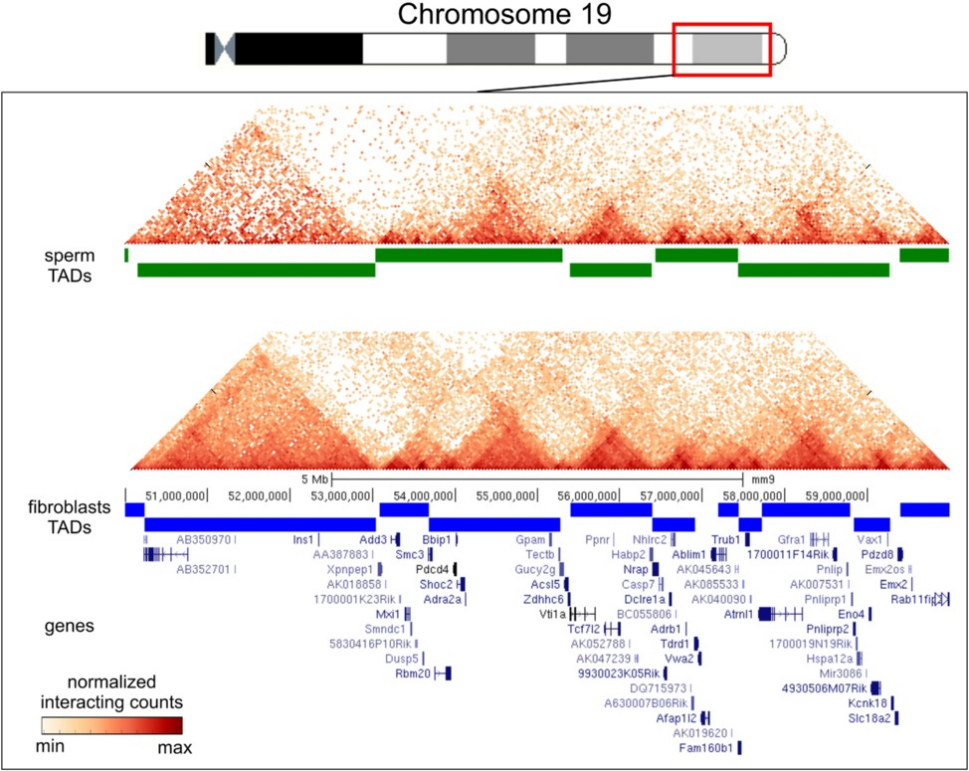
TADs are present in fibroblasts and sperm cells. The TAD signal is shown as a green line (for sperm cells) or a blue line (for fibroblasts) for a region on chromosome 19. The fragments of the heatmaps for sperm cells and fibroblasts (binned at a resolution of 40 kb) display the enrichment of contacts inside the TAD domains. The TAD signal shows visible similarity between sperm cells and fibroblasts.

In addition to the presence of A and B compartments, we identified TADs in sperm cells and fibroblasts (Figure 3; Additional file 2). We found 2,590 domains in fibroblasts (with an average size of 928 kb and a median of 680 kb; Additional file 3). The number and size of TADs in fibroblasts were similar to those described earlier for mouse ES cells (2,200 domains with a median of 880 kb). Interestingly, the number of TADs identified in sperm cells was slightly lower (1,856 domains with an average size of 1,226 kb and a median of 1,000 kb; Additional files 4 and 5). We found that some of the domains identified in fibroblasts were ‘merged’ in sperm cells, that is, genomic regions occupied by one domain in sperm cells might be occupied by several (usually two to four) domains in fibroblasts. This could partially explain the fewer chromatin domains in sperm cells with bigger average size.

**Figure 4:**
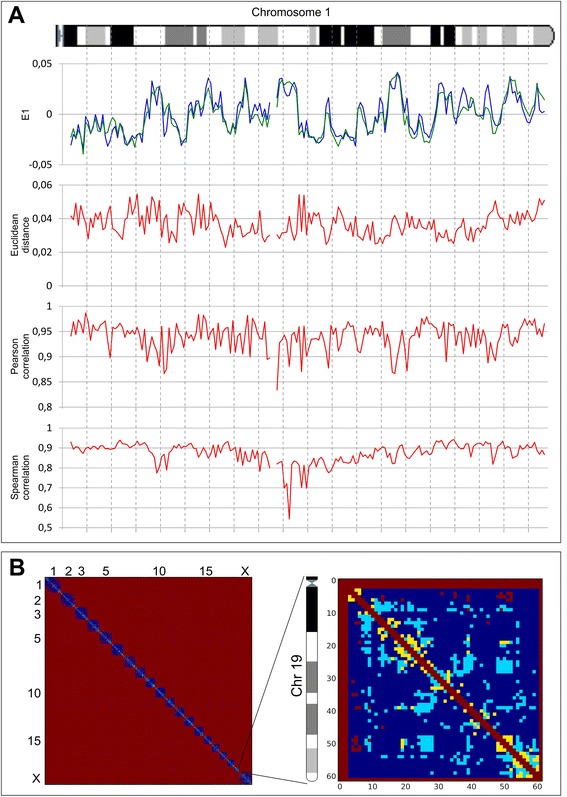
Identification of regions distinguishing sperm cells and fibroblasts. **(A)** E1 values, Euclidean distance and Pearson and Spearman correlation coefficients for chromosome 1 of both sperm cells (green line for E1 values) and fibroblasts (blue line for E1 values). All graphs indicate high similarities between sperm cells and fibroblasts (that is, similar E1 values, small Euclidean distance and high correlation coefficients). However, some regions display less similarity than others. **(B)** The two-dimensional heatmaps for the whole genome and for chromosome 19 (binned at a 1 Mb resolution) indicate the significance of the differences in the contact probability between fibroblasts and sperm cells. Each dot represents a single contact. Regions in red are not mappable, those in yellow are significantly different, those in cerulean are significantly different with a difference of more than two times, and those in blue are contacts where no significant difference was found.

The general similarities of long-range interactions in sperm cell and fibroblast genomes do not exclude dissimilarities in any defined regions. Different analyses, such as the calculation of correlation coefficients, Euclidean distance and comparisons of eigenvector values, have advantages and limitations and could even be connected to different biological properties; therefore, we decided to use all of these approaches together. We first compared the aforementioned characteristics (Pearson and Spearman correlation, Euclidean distance and eigenvector values) for individual bins (Figure 4A). We observed a slight decrease in the Pearson and Spearman correlation coefficients for regions in the middle of chromosome compared with regions near the chromosome end. This difference was due to the statistical insignificance of rare long-range interactions captured for individual bins. We enhanced our comparison method to account for these biases using the self-correlating dataset of ES cells (see Materials and methods for details). Using this method, we selected, in sets of 100, the most different bins for each parameter separately, and we then focused on the bins that were present in all sets. We identified seven bins that greatly differ between fibroblasts and sperm cells (the list of identified genomic regions is shown in Additional file 6). The observed number of bins was more than 10 times higher than expected (approximately 0.1) by a random selection of bins. The identified regions that are dissimilar between sperm cells and fibroblasts are located on chromosomes 5, 12, 13, and 19 (Additional file 6).

We used another approach based on the identification of the individual contacts distinguishing sperm cells and fibroblasts to compare these cells. We considered all contacts supported by more than one read as mappable. Approximately 153,000 (approximately 4.37%) of a potential approximately 3.5 × 10^7^ (at a resolution of 1 Mb) contacts were mappable in sperm and fibroblast genomes. In addition, we found that of the 153,363 mappable interactions for both sperm cells and fibroblasts, 8,947 (5.85%) have significantly different (q-value <0.05) contact probability. Moreover, of these 8,947 interactions, the probabilities of 6,586 contacts showed more than a two-fold difference (Figure 4B). Interestingly, the above-mentioned loci of chromosome 19 show a high amount of significantly different interactions with other regions in the genome (Figure 4B).

**Figure 5:**
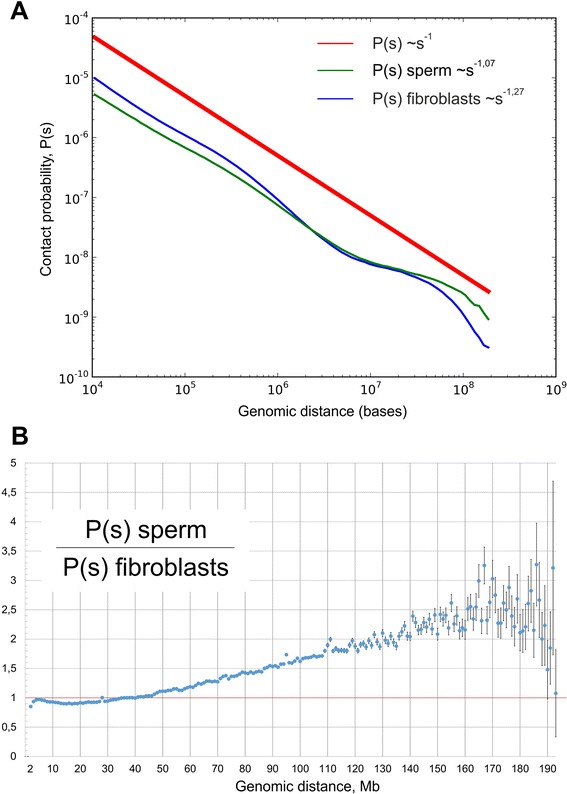
The genome of sperm cells is packed more tightly than that of fibroblasts. **(A)** The dependence of the contact probability on the genomic distance P(s) averaged over all chromosomes, compared with P ~ 1/s. The blue line indicates fibroblasts (P ~ s^-1.27^), and the green line indicates sperm cells (P ~ s^-1.07^). **(B)** The ratio between sperm cells’ and fibroblasts’ contact probabilities at different genomic distances. The x-axis indicates genomic distance, and the y-axis indicates the ratio of contact probabilities. The black lines show a 1:1 ratio.

**Figure 6:**
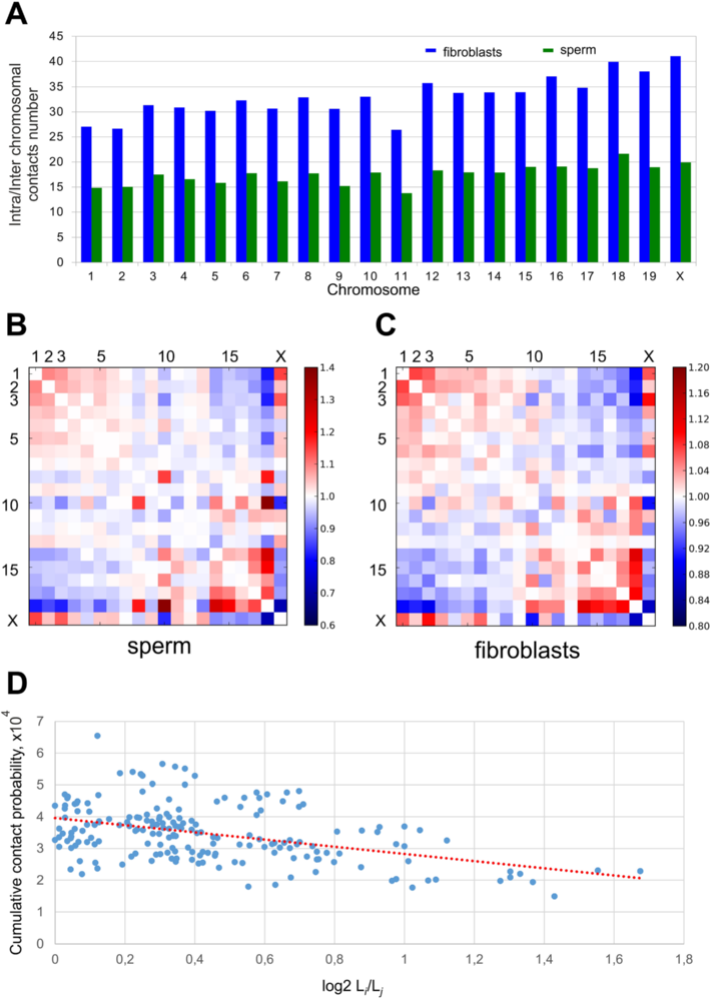
Analysis of intrachromosomal contacts in sperm cells and fibroblasts. **(A)** The ratio between intra- and interchromosomal contact numbers for sperm cells (green) and fibroblasts (blue). **(B,C)** The two-dimensional heatmaps show the observed number of interactions between any pair of chromosomes divided by the expected number of interactions between those chromosomes for sperm cells **(B)** and fibroblasts **(C)**. The color of each dot represents the enrichment (red) or depletion (blue) of contacts compared with the expected values. **(D)** The observed number of interactions between any pair of chromosomes plotted against the difference in the lengths of those chromosomes. The dotted lined represents the linear trend for obtained values.

The dependence of the contact probability of genome loci on the distance between these loci P(s) is informative for understanding the DNA state [10,15,21]. We examined the P(s) dependence in sperm cells and fibroblasts. For both cell types, we observed a strong decrease in contact probability with an increase in the distance between loci, that is, P(s) ~ s^-1.07^ for spermatozoa and P(s) ~ s^-1.27^ for fibroblasts (Figure 5A). The estimated standard errors of the power coefficients at the 95% significance level for both datasets did not exceed 0.01. Thus, the difference between them is significant. They also differ significantly from the ideal fractal model value of −1. However, the packing of the sperm chromosomes appeared more fractal-like than the that of the fibroblast one. Interestingly, the contact probability for fibroblasts was higher, in the range of 10^4^ to 10^6^ bp. This increase in P(s) values was compensated by a lower contact probability in a diapason of long-range interactions at 10^−7^ to 10^−8^ bp. These data suggest that sperm cells have more long-range contacts than do fibroblasts. A detailed analysis showed that the probabilities of contacts in fibroblasts were less than those in sperm cells, when counting regions separated by less than 40 Mb; for loci separated by 50 to 150 Mb, sperm cells display more than two times higher contact probabilities compared with fibroblasts cells (Figure 5B). To better understand how differences in P(s) affect spatial properties of topological features of chromosomes in sperm cells compared with fibroblast cells, we performed modeling of chromatin of these cells. We used BACH [22] to infer the consensus three-dimensional chromosomal structure of sperm cells and fibroblasts. In agreement with a lower amount of long-range interactions in fibroblasts, TADs of these cells appeared to be more ‘elongated’ than compact TADs of sperm cells (Additional file 7). Consistent with an increased amount of long-range interactions, sperm cells display a lower intrachromosomal to interchromosomal contact ratio than fibroblasts (Figure 6A). We observed 25 to 40 times more intrachromosomal contacts than interchromosomal ones in fibroblasts, whereas sperm cells showed only a difference of 12 to 20 times more intrachromosomal contacts. Overall, these data indicate that the genome of spermatozoa is packed more compactly, such that more distant loci are brought together and have a high probability of contact with each other.

In the generated heatmaps, individual chromosomes showed up as compact contact-enriched clusters (Figure 1). Indeed, more than 90% of the interactions fell into intrachromosomal contacts in both sperm and fibroblast cells. Statistical analyses of rare interchromosomal contacts revealed that chromosomes are distributed non-randomly in sperm and fibroblast nuclei (Figure 6B). The whole chromosome interaction patterns show that the large chromosomes (for instance, chromosomes 1 to 8 and X) are more likely to interact with one another and not with the small chromosomes (chromosomes 14 to 19), while the shorter chromosomes show a tendency to establish contact with each other (Figure 6B). A similar pattern of interactions between chromosomes was identified in fibroblasts (Figure 6C). This observation was further confirmed by an opposite correlation between probabilities of contacts between chromosomes and differences in their lengths (Pearson r = −0.44; Figure 6D). These data are in agreement with the previously published Hi-C results [13].

The differences between the three-dimensional organizations of sperm cells and fibroblasts can potentially originate from two independent sources. First, the denser packaging of the sperm genome compared with the fibroblast genome is due to a decrease in the nucleus size and a denser packaging of DNA with protamines compared with histones. Second, local rearrangements of three-dimensional genome structures are due to the loss or gain of functional connections between different loci. To estimate the role of the first reason (that is, the denser packaging of the sperm genome), we developed a normalization process referred to as the ‘compression’ of the fibroblast genome to a sperm cell’s parameters. Our normalization does not change the distribution of contact probabilities for regions separated by the same genomic distance but instead brings all loci closer to each other. Thus, we normalized the number of fibroblast contacts between loci separated by a given genomic distance to achieve the same P(s) distribution for fibroblasts and sperm cells, but we maintained the contact ratios of loci separated by the same genomic distance. We compared the obtained post-‘compression’ fibroblasts (C_Sp_-fibroblasts) with the sperm cells and found that the number of contacts that had different probabilities between sperm cells and fibroblasts decreased from 8,974 to 6,962 after compression (Additional file 8). Additionally, the number of contacts with more than a two-fold difference in contact probabilities decreased from 6,586 to 5,009. As a control, we performed the compression of fibroblasts to ES cell parameters, thereby obtaining C_ESC_-fibroblasts. The number of differences in contact probabilities between C_ESC_-fibroblasts and sperm cells increased up to 10,848, with more than 8,776 contacts showing at least a two-fold difference. However, the C_ESC_-fibroblasts were more similar to ES cells than original fibroblasts were to ES cells, indicating that compression decreases differences only when performed in a cell-specific manner. Our data imply that approximately 25% of the differences in contact probabilities between sperm cells and fibroblasts might originate from differences in the densities of their genome packaging; however, the main source of differences is the gain or loss of contacts that are specific to defined genome regions.

## Discussion

The obtained data are the first description of three-dimensional organization in mouse motile sperm cells and fibroblasts obtained using Hi-C technology. Though spermatozoa and fibroblasts are extremely different in a number of aspects, the spatial organization of DNA in these cells is similar. Moreover, two types of previously identified domains, that is, A and B compartments [10,15] and TADs [16], were present in both sperm and fibroblast genomes.

The high similarity of E1 values for sperm cell data produced by TCC and cortex data produced by Hi-C suggests that this correlation is due to similar folding of chromosomes in these two contexts, as opposed to potential Hi-C- or TCC-specific biases. Indeed, the correlation between E1 values of sperm cells and cortex data was even slightly higher than between sperm cells and fibroblasts, despite the first being produced using different methods (Hi-C and TCC) whereas the last were produced using a similar TCC protocol. This is in agreement with the close similarity (R > 0.95) of Hi-C maps produced by the ‘classical’ Hi-C method (described in [10]), a TCC-based method (described in [13]) and a novel *in situ* Hi-C method (described in [23]), at least at a resolution of 100 kb and above, observed in other studies [13,23].

It is still unknown whether the presence of spatial domains in cells is an indirect result of DNA packaging in nucleosomes and the transcription process or whether there are special mechanisms involved in the formation and maintenance of spatial domains. In sperm cells, DNA packaging is influenced at a very basic level by the replacement of histones with different proteins, that is, protamines; the transcription process is also completely abolished [18]. However, the high-order chromatin structure of the cell remains stable. This finding suggests the presence of special mechanisms involved in the establishment and maintenance of these structures and highlights an important role for spatial domains in cell function.

Despite the remarkable similarity of the three-dimensional genome organization between sperm cells and fibroblasts, we aimed to find regions that distinguish these cell types. We used three independent methods (Pearson correlation, Euclidean distance and eigenvector comparison) to compare the three-dimensional organization of the genomes of sperm cells and fibroblasts. Some of these methods (Pearson correlation and eigenvector comparison) have been used previously [10,15]; we introduced Euclidean distance as a method for comparing individual genomic regions. Though the overlap between the sets of genomic regions obtained using different methods was more than 10 times larger than expected for randomly selected regions, it was still far from 100%. One explanation for this result could be a difference in the sensitivities of the methods used to estimate systematic biases of the Hi-C experiment. Another, more intriguing explanation is that the different mathematical methods used to compare individual regions reflect different biological properties of these regions. Additional studies are required to develop a standardized approach for the comparison of several Hi-C datasets.

To explain the differences observed in sperm cells and fibroblasts, we developed a normalization method accounting for genome compression. Our normalization shows that genome compression can explain approximately 30% of the differences between sperm cells and fibroblasts. The further development of such a model might allow understanding the changes in the three-dimensional structure of chromatin from a new point of view.

The differences described above, that is, in the long-range contacts in sperm cells and fibroblasts, are most likely related to the extreme compactness of the sperm genome. The increased average size of TADs in sperm cells compared with fibroblasts, as well as the fact that some domains are ‘merged’ in sperm cells, support this suggestion. However, one should note that identification of TADs is a matter of the mathematical algorithm and parameters used for calling TADs. We employed the most commonly used algorithm and parameters for TAD calling to make our data comparable with other reports [16,24-26]. However, this algorithm does not allow identification of subdomains, that is, alternative sets of small domains located at defined regions of the genome. Thus, we cannot exclude that TADs identified in the fibroblast genome are also present in sperm cells, but are not fully visible to the TAD calling algorithm due to the increase in long-range contacts.

Finally, the possible explanation for the aforementioned differences in TADs could originate from cell type-specific functional looping. For instance, the genome of mouse cortex contains 1,519 domains with an average size of 1.54 Mb and a median of 1.32 Mb, which is different from ES cells and fibroblasts but even more close to the parameters observed in sperm cells. Thus, the functional role of the number and average size of TADs with respect to particular cell types remains to be elucidated.

Extreme compactness of the sperm genome might also be a reason for the increased frequency of interchromosomal interactions, resulting in different intrachromosomal to interchromosomal contact ratios in sperm cells and fibroblasts. In fact, the DNA within the sperm nucleus is packed in a volume that is approximately 5% of the volume in somatic cells [18]. Here, it is pertinent to note that the compactness of the sperm genome is comparable to that of metaphase chromosomes. Recently, Naumova *et al*. [21] reported a homogenous folding state that is locus-independent and common to all chromosomes at their metaphase status in examined cell types. Keeping in mind the similarity of the three-dimensional organization of sperm cells and fibroblasts, one could suggest that the exceptional compactness of the sperm genome is not sufficient in itself to change hierarchical models of chromatin structure [11,15,16].

On the other hand, some of the specific looping identified in this study can be caused by the difference between fibroblast and primordial germ cells, which do not reflect the genome compaction during spermatogenesis. Additional studies of primordial germ cells are required to resolve this possibility.

We found the P(s) distribution in sperm cells is closer to the fractal-like model of genome organization than in fibroblasts. The P(s) distributions in spermatozoa and fibroblasts were strictly different from those found in mitotic chromosomes [21], emphasizing differences in the mechanisms of genome compression during mitosis and sperm maturation.

In summary, the remarkable similarities in the three-dimensional genome organization of spermatozoa and fibroblasts show the role of male gametes as carriers of the three-dimensional genome organization through generations.

## Conclusions

Taken together, our findings suggest that genomic spatial contacts are (largely) consistent across the sperm cells and spermatozoa with >90% of interactions being seen in both cell types. However, there are specific dissimilar regions, that is, on chromosomes 5, 12, 13, and 19. Spermatozoa have more long-range contacts than fibroblasts, which makes sense considering their nuclei are more compact, and approximately 30% of the differences in interaction probabilities between the two cell types can be explained by differences in the density of their genome packaging.

## Materials and methods

### Ethics statement

All animal protocols were approved by the Ethical Committee of the Institute of Cytology and Genetics (protocol number 17.4_17.06.2013).

### Preparation of motile sperm cells and mouse embryonic fibroblasts

Mature mouse spermatozoa were obtained from the epididymis of C57BL mice using the swim-up assay [27]. Briefly, *cauda epdidymis* was dissected into pieces and placed into sperm motility medium (135 mM NaCl, 5 mM KCl, 1 mM MgSO_4_, 2 mM CaCl_2_, 30 mM Hepes, pH 7.4; freshly supplemented with 10 mM lactate acid, 1 mM sodium pyruvate, 20 mg/ml bovine serum albumin, 25 mM NaHCO_3_) for 1 h at 37°C. To avoid contamination by somatic cells, only the top fractions containing motile sperm were collected. The cell suspension was centrifuged, and the cell pellets were resuspended in serum free Dulbecco’s modified Eagle’s medium (DMEM) and processed for Hi-C library generation as described below.

Mouse embryonic fibroblasts were obtained from 13-day-old embryos from C57BL mice and cultured in standard conditions, as described previously [28].

### Generation of Hi-C libraries

Hi-C libraries were produced using a TCC protocol [13], but with some minor modifications. Briefly, 50 million sperm cells were resuspended in 45 ml serum free DMEM, and 37% formaldehyde was added to obtain a final concentration of 1% for cross-link chromatin. Mouse fibroblasts were fixed while they were attached to the culturing surface in 1% final concentration of formaldehyde in the serum-free DMEM. Cells were incubated at room temperature for 10 minutes; the formaldehyde was then quenched by adding glycine to obtain a final concentration of 0.125 M, and the mixture was incubated at room temperature for 5 minutes and subsequently on ice for 15 minutes. Mouse fibroblasts were scraped from the culture plate using disposable cell scrapers and aliquoted for 25 million cells. Sperm cells were harvested by centrifugation. After crosslinking, the sperm and fibroblast samples were processed identically. Fixed cells were lysed using a Dounce homogenizer in the presence of cold lysis buffer (10 mM HEPES, pH 8.0, 10 mM NaCl, 0.2% IGEPAL CA-630, and 1× protease inhibitor solution).

The chromatin was solubilized with dilute sodium dodecyl sulfate (SDS) and incubated at 65°C for 10 minutes. The chromatin was biotin labeled chemically by EZ-Link-Iodoacetyl-PEG2-biotin (Pierce Protein Research Products, Rockford, Illinois, USA). DNA in the cross-linked protein complexes was digested with HindIII endonuclease. Biotinylated digested chromatin was immobilized on MyOne Streptavidin T1 beads (Invitrogen, Grand Island, New York, USA). The 5′ overhang was filled in by the Klenow fragment of DNA polymerase I using equimolar amounts of all deoxyribonucleotides, with the substitution of biotin-14-dCTP for dCTP. The immobilized blunt-ended DNA fragments were then ligated while they were tethered to the surface of the beads. The chromatin complexes containing the biotin-labeled ligation products were degraded by incubation with Proteinase K at 65°C. DNA was purified by phenol-chloroform extraction. The biotinylated nucleotide was removed from non-ligated DNA ends using T4 DNA polymerase, as previously described [29]. The DNA was sheared and size-selected; the fragments that included a ligation junction were then isolated on streptavidin-coated magnetic beads and prepared for paired-end sequencing. The libraries were sequenced on an Illumina Genome Analyzer IIx (GA IIx) machine using the paired-end module and with 50 bp reads on each end.

### Generation of heatmaps

Sequencing reads were mapped to the mm9 mouse genome and filtered using the pipeline developed by Imakaev *et al*. [15]. Mirnlib version 0d30147f052f and hiclib version d28d8d985120 software were obtained from [30]. The public datasets SRR443883, SRR443884 and SRR443885 [16] were processed similarly to obtain Hi-C data for mouse ES cells. Heatmap computation, iterative correction, eigenvector decomposition and P(s) calculation were performed using the hiclib software [15]. For each interaction, we estimated an error as $\frac{\sqrt{K}}{K}$, where K is a number of reads supporting the interaction. The average error for non-zero intrachromosomal interactions in sperm cells was 24% at the 1 Mb scale and reached 88% on a 0.1 Mb scaled heatmap. Therefore, we used the 1 Mb resolution for all subsequent calculations, except cases where the resolution is specifically indicated. The two-dimensional contact correlation matrices were constructed as in [10].

### Identification of regions different between sperm cells and fibroblasts

We calculated the Euclidean distance, Pearson correlation and eigenvector differences between individual bins of sperm and fibroblasts cells.

We calculated Euclidean distance between individual bins as:

$$ E=\sqrt{{\displaystyle \sum_{j={c}_{st}..{c}_{end},j\ne i}}\ {\left(S{p}_{ij}-Fi{b}_{ij}\right)}^2} $$

where E is the Euclidean distance between bin ‘i’ at chromosome chr_i_, c_st_ is the first bin of chromosome chr_i_, c_end_ is the last bin of chromosome chr_i_, and Sp_ij_ and Fib_ij_ are the number of reads supporting contacts between bins ‘i’ and ‘j’ of sperm cells and fibroblasts. A greater Euclidean distance indicates a larger difference between bins.

The Pearson and Spearman correlation coefficients were calculated for each bin, accounting only for intrachromosomal contacts. The signal-to-noise ratio might vary in a Hi-C experiment, even if the data are iteratively corrected. The enrichment of any genomic region with interactions that have low signal-to-noise ratios might result in an underestimation of the correlation coefficients for these genomic regions. To handle this problem, we used the correlation of two random sub-datasets (‘reference’ datasets) generated from the ES cells dataset as a marker for regional-dependent biases. We defined poor bins as those that satisfy the condition:

$$ {\mathrm{C}}_{\mathrm{i}} < \mathrm{M} - \mathrm{S}\mathrm{D} $$

where C_i_ is the Pearson or Spearman correlation coefficient between bin ‘i’ in the ‘reference’ datasets, and M and SD are the median and standard deviation of the Pearson correlation coefficient for all bins in the ‘reference’ datasets. We observed that C_i_ values for one bin upstream and downstream of poor regions were also strongly less than average (Additional file 9). Based on this observation, we excluded from the analysis all poor bins as well as one bin both upstream and downstream of each poor bin. Because the Spearman correlation coefficient is rank-based, it is more sensitive to small differences in the samples observed at low signal-to-noise ratios. We therefore used the Pearson correlation coefficient for the subsequent analysis.

The first eigenvector (E1) values of the sperm and fibroblast cells were calculated using the hiclib software, as described previously [15]. We considered each bin to be a dot, with the X-coordinate equal to the appropriate E1 value of sperm cells and the Y-coordinate equal to the appropriate E1 value of fibroblasts. We computed a linear regression line for the obtained dots using the least-squares method. We than calculated a distance from each dot to the regression line and used this distance as a measure of the difference between the eigenvectors of the appropriate bins. A greater distance indicates a larger difference between bins. Additionally, a maximal information coefficient was calculated for E1 values for each pair of datasets as described in [20], using MINE python implementation [31] with default parameters (alpha = 0.6, c = 15).

We ranked all bins in the sperm cell and fibroblast datasets using three types of ranks (ranks generated during the calculations of Euclidean distance, Pearson correlation and eigenvectors difference). From the 2,308 bins, we selected the 100 highest-ranked bins for each type of analysis and defined them as candidate bins. This process resulted in three sets of candidate bins. Some of the candidate bins might have had high ranks due to region-specific biases (for example, described in the calculation of Pearson correlation coefficient). To exclude such regions, we calculated the Euclidean distance and the difference between the eigenvectors for two ‘reference’ ES cell datasets (described above in the calculation of Pearson correlation coefficient), selected the 100 highest-ranked bins for each type of analysis and excluded them from the candidate bins. Finally, we identified regions that were present in all three sets’ candidate bins and defined them as regions that differed between sperm cells and fibroblasts. The expected number of regions that differed between sperm cells and fibroblasts was calculated as:

$$ \frac{N_{Eucl}*{N}_P*{N}_{E1}}{{N_T}^2} $$

where *N*_*Eucl*_, *N*_*P*_, *N*_*E*1_ are the number of bins remaining after filtering candidate bins with ranks according to Euclidean distance (*N*_*Eucl*_), Pearson correlation (*N*_*P*_) and eigenvector difference (*N*_*E1*_) and *N*_*T*_ is the total number of bins (2,308).

### Identification of differences in individual contact probabilities in sperm cells and fibroblasts

We used a uniform probability model to describe the contact frequencies observed in Hi-C experiments [32]. Assuming that the probability of observing any particular interaction is uniform, the probability of contacts between bins ‘i’ and ‘j’ (*P*^*i*,*j*^) is:

$$ {P}^{i,j}=\frac{m}{M} $$

where m is the number of reads supporting the interaction (normalized in the iterative correction) and M is the total number of reads (normalized in the iterative correction). Note that when counting reads, we only considered contacts that were supported by more than one read (mappable contacts). We used following criteria of normal approximation of binomial distribution: M × P^i,j^ × (1 - P^i,j^) > 9 [33] and excluded all contacts that do not satisfy the criteria. We tested the null hypothesis ${H}_0:\ {P}_{Sp}^{i,j}={P}_{Fib}^{i,j}$, where ${P}_{Sp}^{i,j},{P}_{Fib}^{i,j}$ are the probabilities of contacts between bins ‘i’ and ‘j’ in sperm cells (P_Sp_) and fibroblasts (P_Fib_). Assuming normal approximation of binomial distribution, we calculated the *P*-value for the null hypothesis as:

$$ {p}^{i,j}=2* Norm\left(\frac{\ {P}_{Sp}^{i,j}-{P}_{Fib}^{i,j}}{\sqrt{\frac{\ {P}_{Sp}^{i,j}*\left(1 - {P}_{Sp}^{i,j}\right)}{M_{Sp}} + \frac{P_{Fib}^{i,j}*\left(1 - {P}_{Fib}^{i,j}\right)}{M_{Fib}}}}\right)-1 $$

where *Norm* is the normal distribution. We than calculated q-values by multiplying each *P*-value by the total number of hypotheses tested.

### Modeling of fibroblast genome ‘compression’

To perform the fibroblast genome ‘compression’, we first calculated compression coefficients K_j_ as:

$$ {K}_j = \frac{S{p}_j}{Fi{b}_j} $$

where Sp_j_ and Fib_j_ represent the sum of elements of diagonal j in an iteratively corrected Hi-C matrix for sperm cells or fibroblasts, respectively. Errors for coefficient calculation were estimated in the same way as described above (see the ‘Generation of heatmaps’ section). We then performed a correction by multiplying all contacts at the diagonal j of the fibroblast datasets by the appropriate coefficient compression coefficients. We did not apply a correction if the coefficient error was above 5%. Finally, we adjusted all contacts to achieve the same total sum of elements for both the resulting and original matrices.

The chromosome interaction patterns were calculated as described previously [10,13]. Briefly, the observed/expected contact frequencies for chromosomes ‘i’ and ‘j’ were calculated as:

$$ \frac{S_{ij}}{\left({S}_i*\frac{S_j}{T-{S}_i}+{S}_j*\frac{S_i}{T-{S}_i}\right)*0.5} $$

where S_i_ and S_j_ are the sum of interchromosomal contacts of chromosomes ‘i’ and ‘j’, respectively, S_ij_ is the sum of contacts between chromosomes ‘i’ and ‘j’, and T is the total sum of all interchromosomal contacts.

### Identification of topologically associated domains

To identify TADs, heatmaps were binned at 40 kb resolution, iteratively corrected and analyzed using a previously developed pipeline [16].

### Chromatin modeling

Chromatin modeling was performed using the BACH algorithm [22] with default parameters. The get Annotated Restriction Sites function from the HiTC package [34] was used to compute the mappability score for each restriction site in the mm9 genome, and Mirnlib was used to calculate GC percentage and number of restriction sites for each locus analyzed. Each TAD identified in fibroblasts and sperm cells was processed separately. The resulting posterior mode of the three-dimensional coordinates was used to calculate the HD-ratio of TADs as described in [22]. The HD-ratio was used as a measure of TAD compactness, that is, domains with a higher HD-ratio were assumed to be more elongated and less compact. The normalized HD-ratio (HD-ratio divided by the TAD length) was introduced to account for differences in TAD length as in [22]. Mann–Whitney test was used to compare the HD-ratio of sperm cells and fibroblasts, and *P*-value <0.001 was considered as a threshold for statistically significant differences.

### Data availability

The sequencing results of Hi-C libraries of sperm cells and fibroblasts are available in the NCBI Sequence Read Archive under accession number SUB540202 (SRX553176 for sperm cell data and SRX554530 for fibroblast data).

## Additional files

Additional file 1:
**Spearman correlation and maximal information coefficients between E1 values of sperm cells, fibroblasts, ES cells and cortex.**
Additional file 2:
**TADs identified in fibroblasts and sperm cells display similar, but not equal, distribution.** The TAD signal is shown as a green line (for sperm cells) or a blue line (for fibroblasts) for a region on chromosome 3. In some regions TADs are different (for example, the most left TAD, region A), in some similar (for example, the most right TAD, region C) and in some ‘nested’ (for example, TADs in the middle of the region, region B). Additional file 3:
**TADs identified in fibroblasts.** Coordinates of TADs identified in fibroblasts in USCS bed-track format. Additional file 4:
**TADs identified in sperm cells.** Coordinates of TADs identified in sperm cells in USCS bed-track format. Additional file 5:
**Sperm cell TADs display higher average size.** Box-and-whisker plot showing TAD sizes in fibroblasts and sperm cells. Additional file 6:
**Localization of seven regions on a genome map, as identified by overlapping different datasets of highly dissimilar regions between sperm cells and fibroblasts.**
Additional file 7:
**TADs of fibroblasts are more ‘elongated’ than sperm cell TADs.** (A,B) Normalized (A) and not normalized (B) HD-ratios (see Materials and methods for details of HD-ratio calculation) of fibroblasts and sperm cell TADs are presented as standard errors of the mean. Asterisks indicate significance of differences. Additional file 8:
**Estimation of number of contacts distinguishing sperm and other cell types.** The bar plot shows total number of interactions in 1 Mb-binned heatmaps (in 1,000-fold scale), number of ‘non-zero’ interactions (that is, all mappable contacts for satisfying criteria of normal approximation of binomial distribution, see Materials and methods for details), and number of interactions with different frequencies (q-value <0.05) obtained when comparing sperm cells, fibroblasts or ES cell datasets and ‘compressed’ derivations of these datasets. Additional file 9:
**Nearest to poor bins regions show strong decrease in average Pearson correlation of ‘reference’ datasets.** The graph shows average Pearson correlation of ‘reference’ datasets (see ‘Identification of regions different between sperm cells and fibroblasts’ subsection of Materials and methods for definition of ‘reference’ datasets) plotted against distance from poor bins.

## Abbreviations

bp: base pair
DMEM: Dulbecco’s modified Eagle’s medium
ES: embryonic stem
TAD: topologically associated domain
TCC: tethered conformation capture.
