# Erratum to: comparison of the three-dimensional organization of sperm and fibroblast genomes using the Hi-C approach

**DOI:** 10.1186/s13059-016-0868-5

**Published:** 2016-01-14

**Authors:** Nariman Battulin, Veniamin S. Fishman, Alexander M. Mazur, Mikhail Pomaznoy, Anna A. Khabarova, Dmitry A. Afonnikov, Egor B. Prokhortchouk, Oleg L. Serov

**Affiliations:** Institute of Cytology and Genetics, Novosibirsk, 630090 Russia; Novosibirsk State University, Novosibirisk, 630090 Russia; Center ‘Bioengineering’, Russian Academy of Sciences, Moscow, 123098 Russia; National Research Center, Kurchatov Institute, Moscow, 123098 Russia; Skoltech Center for Stem Cell Research, Skolkovo Institute of Science and Technology, Skolkovo 1443025, Moscow, Russia

It has come to our attention that there are a number of minor errors in our recent article [1].

1. In the sentence ‘This increase in P(s) values was compensated by a lower contact probability in a diapason of long-range interactions at 10^−7 to 10^−8 bp.’, the negative signs should be removed. That is, it is a lower probability of long-range interactions at 10^7 to 10^8 bp.
2. In the sentence ‘A detailed analysis showed that the probabilities of contacts in fibroblasts were less than those in sperm cells, when counting regions separated by less than 40 Mb; for loci separated by 50 to 150 Mb, sperm cells display more than two times higher contact probabilities compared with fibroblasts cells (Fig. 5b).’ it should say ‘more than those in sperm cells’.
3. There is an error in Fig. 6, panels b and c. In the original figure, the labeling of the rows and columns on the heatmaps got misaligned, such that row/column 6 was labeled 5, 11 was labeled 10, and so on. A corrected version of the figure is provided. So for instance, there is a strong contact between chromosomes 9 and 11, and not between 10 and 12 as it appeared on the earlier version. The legend of the figure is unchanged.

**Fig. 6:**
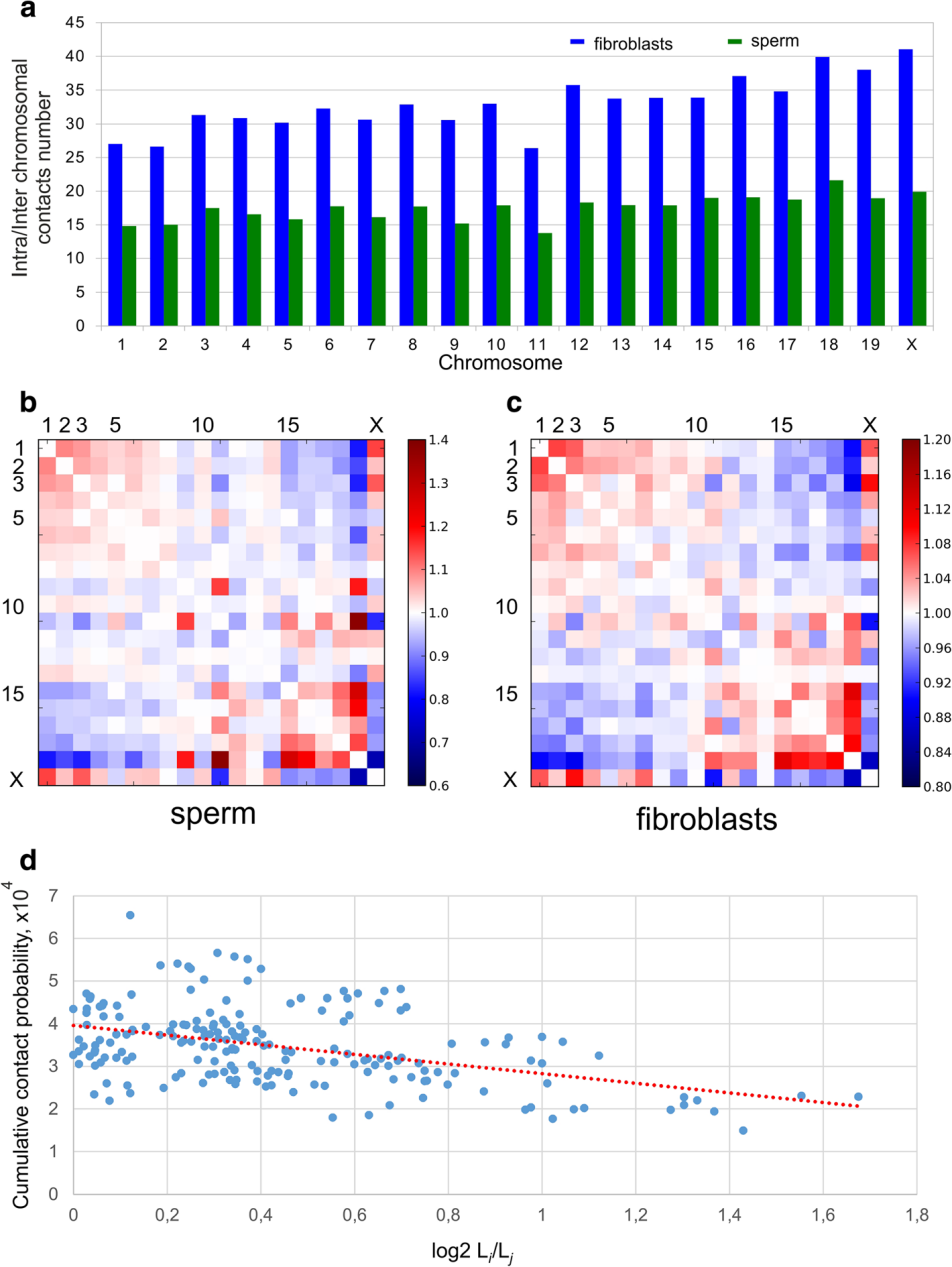
Analysis of intrachromosomal contacts in sperm cells and fibroblasts. **a** The ratio between intra- and interchromosomal contact numbers for sperm cells (green) and fibroblasts (blue). (**b**, **c**) The two-dimensional heatmaps show the observed number of interactions between any pair of chromosomes divided by the expected number of interactions between those chromosomes for sperm cells (**b**) and fibroblasts (**c**). The color of each dot represents the enrichment (red) or depletion (blue) of contacts compared with the expected values. **d** The observed number of interactions between any pair of chromosomes plotted against the difference in the lengths of those chromosomes. The dotted lined represents the linear trend for obtained values
