# Supplementary material for: Comparison of the three-dimensional organization of sperm and fibroblast genomes using the Hi-C approach
Source: Genome Biol. 2015 Apr 14;16(1):77. doi: 10.1186/s13059-015-0642-0 (PMC4434584; doi:10.1186/s13059-015-0642-0)
Supplement: Additional file 1: — Spearman correlation and maximal information coefficients between E1 values of sperm cells, fibroblasts, ES cells and cortex. [file 13059_2015_642_MOESM1_ESM.docx]

Additional file 1

Spearman correlation coefficients between E1 values of sperm cells, fibroblasts, ESC and cortex

|  | Sperm cells | Fibroblasts | Cortex | ESC |
| --- | --- | --- | --- | --- |
| Sperm cells | 1,000 | 0,899 | 0,901 | 0,878 |
| Fibroblasts | 0,899 | 1,000 | 0,823 | 0,821 |
| Cortex | 0,901 | 0,823 | 1,000 | 0,893 |
| ESC | 0,878 | 0,821 | 0,893 | 1,000 |

Maximal information coefficients measured for E1 values of sperm cells, fibroblasts, ESC and cortex

|  | Sperm cells | Fibroblasts | Cortex | ESC |
| --- | --- | --- | --- | --- |
| Sperm cells | 1,000 | 0,673 | 0,693 | 0,651 |
| Fibroblasts | 0,673 | 1,000 | 0,567 | 0,575 |
| Cortex | 0,693 | 0,567 | 1,000 | 0,683 |
| ESC | 0,651 | 0,575 | 0,683 | 1,000 |
