# Supplementary figures and images for: Comparison of the three-dimensional organization of sperm and fibroblast genomes using the Hi-C approach
Source: Genome Biol. 2015 Apr 14;16(1):77. doi: 10.1186/s13059-015-0642-0 (PMC4434584; doi:10.1186/s13059-015-0642-0)

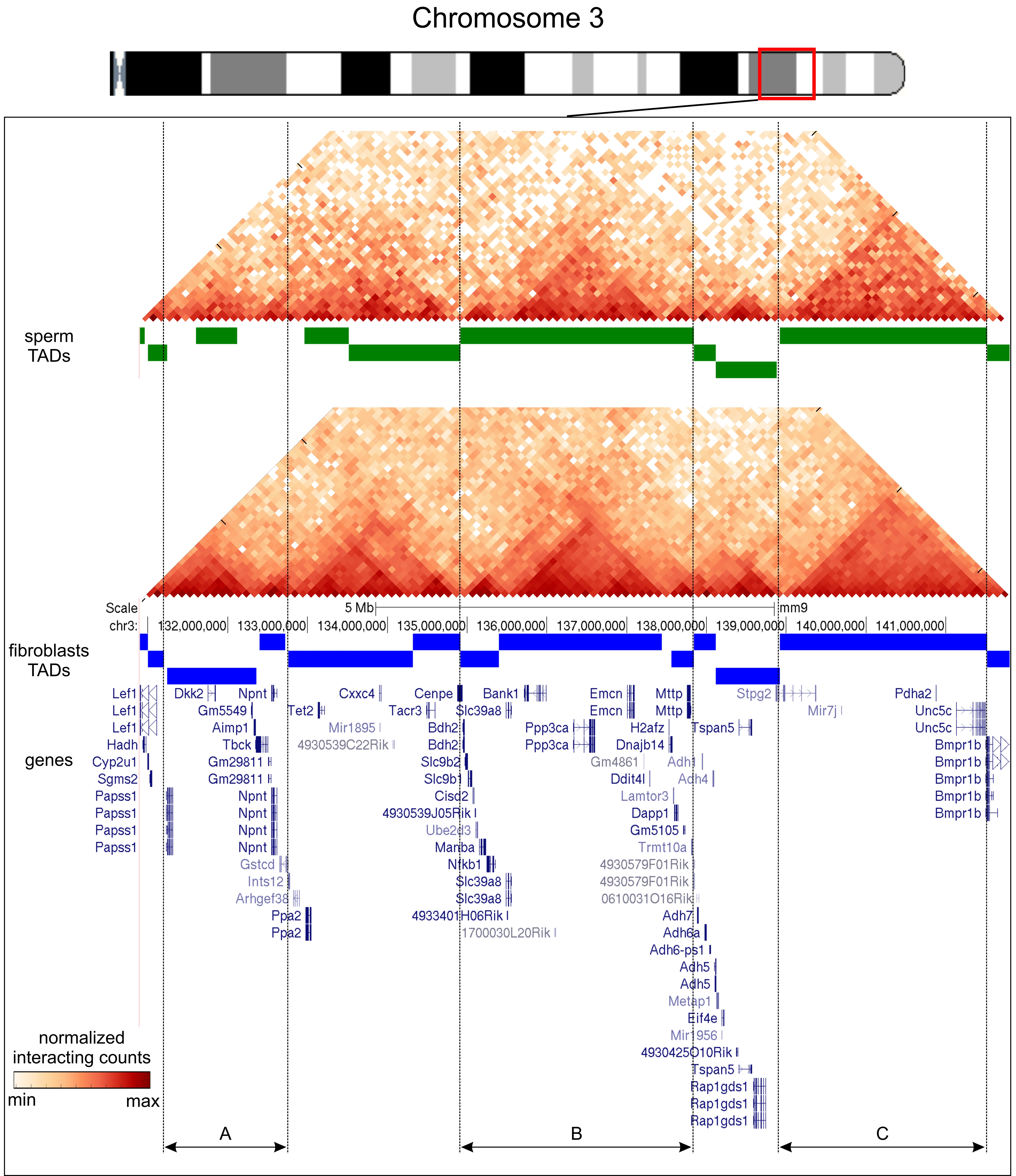

Supplement: Additional file 2: — TADs identified in fibroblasts and sperm cells display similar, but not equal, distribution. The TAD signal is shown as a green line (for sperm cells) or a blue line (for fibroblasts) for a region on chromosome 3. In some regions TADs are different (for example, the most left TAD, region A), in some similar (for example, the most right TAD, region C) and in some ‘nested’ (for example, TADs in the middle of the region, region B). [file 13059_2015_642_MOESM2_ESM.jpeg]

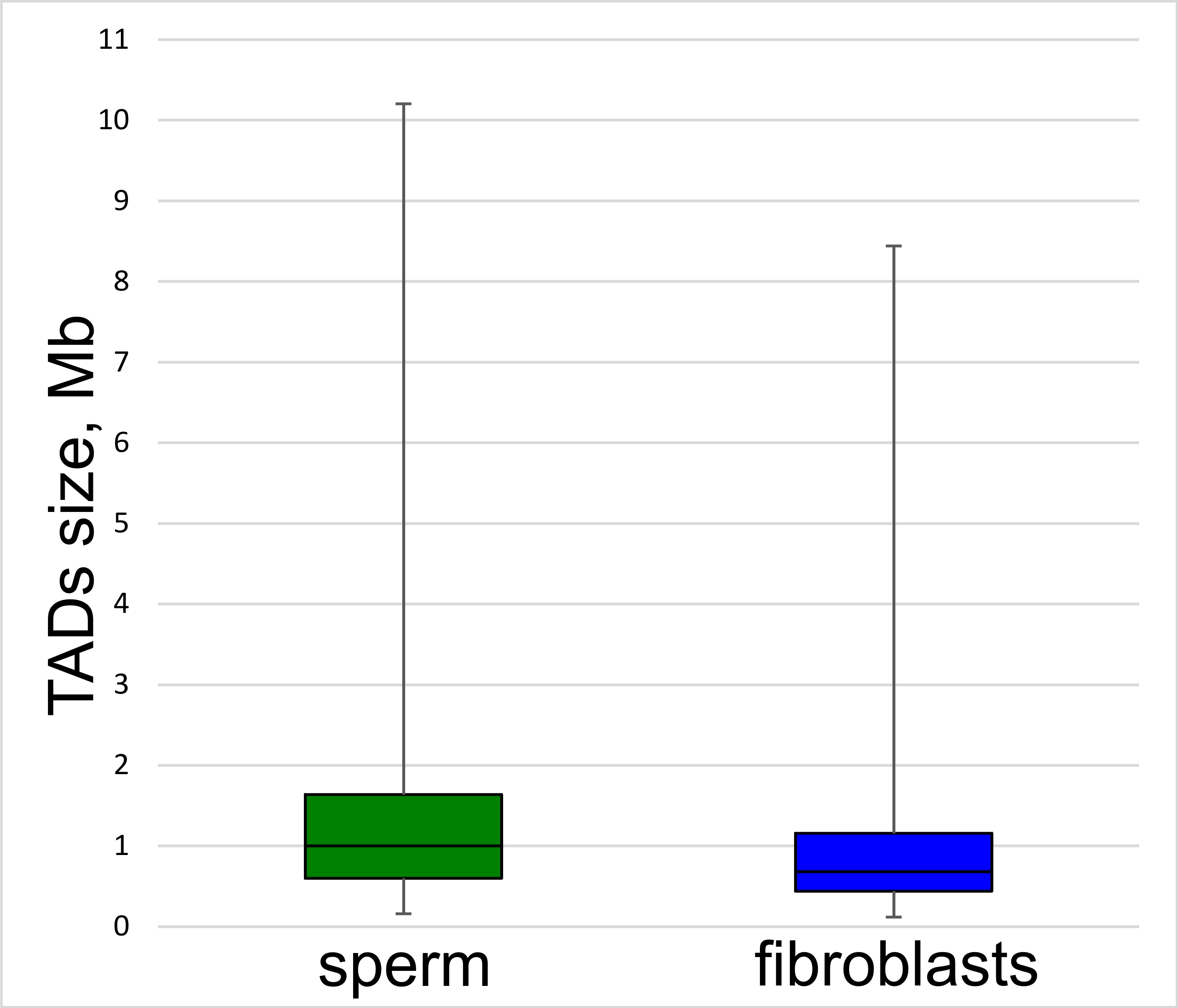

Supplement: Additional file 5: — Sperm cell TADs display higher average size. Box-and-whisker plot showing TAD sizes in fibroblasts and sperm cells. [file 13059_2015_642_MOESM5_ESM.jpeg]

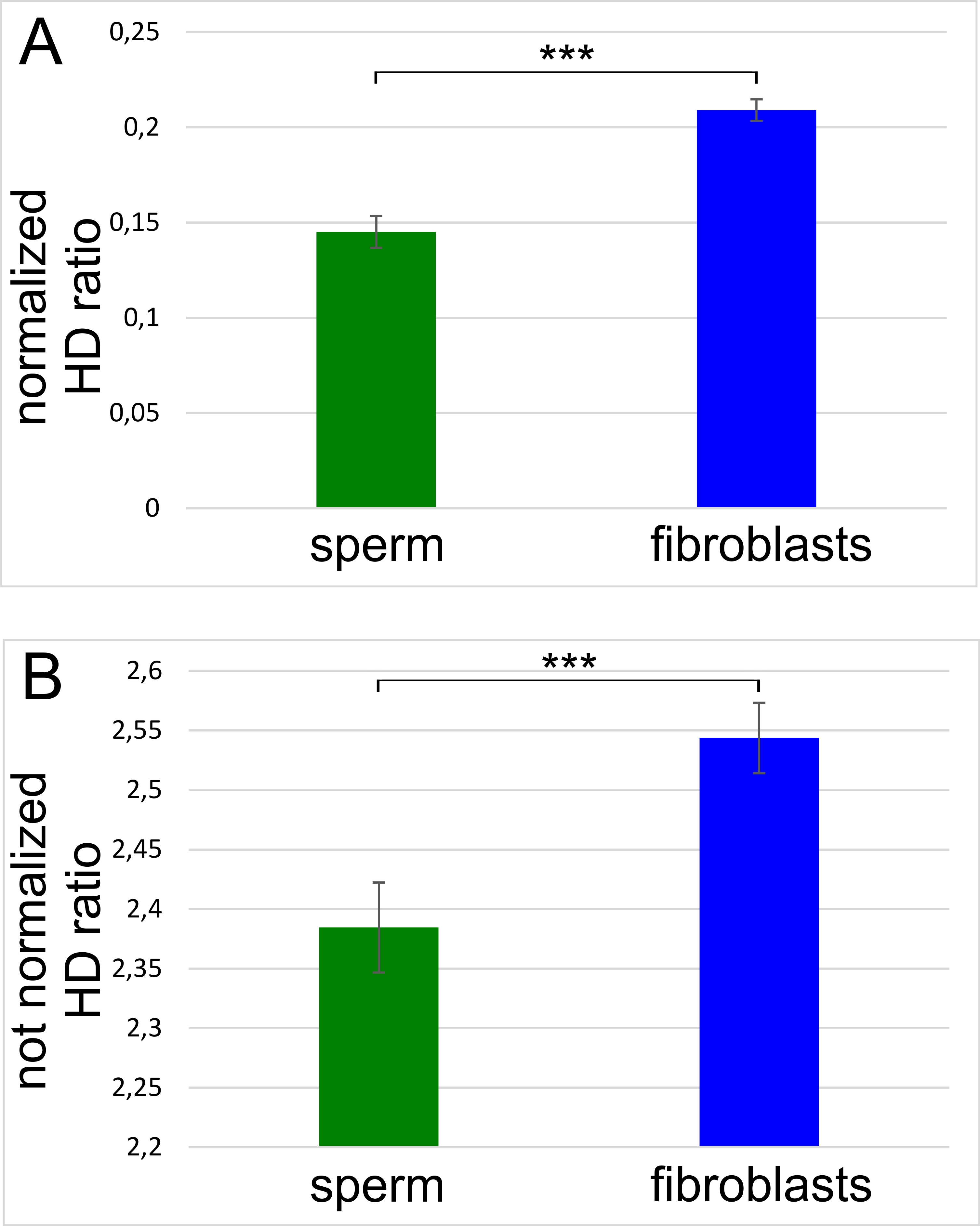

Supplement: Additional file 7: — TADs of fibroblasts are more ‘elongated’ than sperm cell TADs. (A,B) Normalized (A) and not normalized (B) HD-ratios (see Materials and methods for details of HD-ratio calculation) of fibroblasts and sperm cell TADs are presented as standard errors of the mean. Asterisks indicate significance of differences. [file 13059_2015_642_MOESM7_ESM.jpeg]

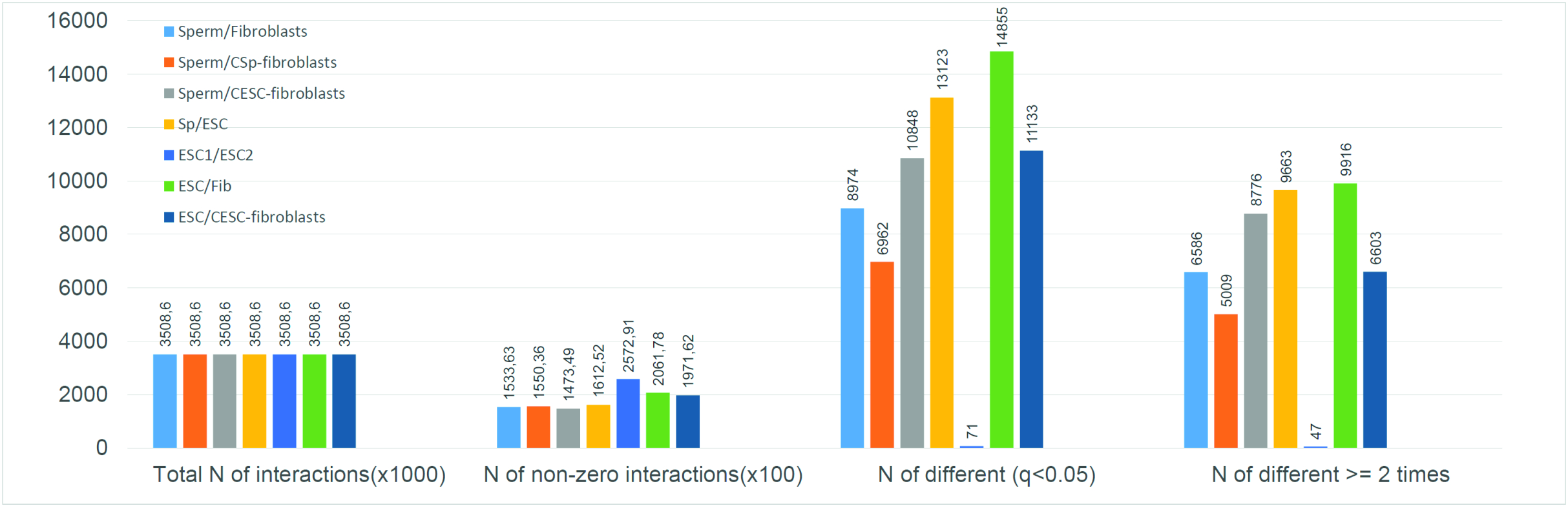

Supplement: Additional file 8: — Estimation of number of contacts distinguishing sperm and other cell types. The bar plot shows total number of interactions in 1 Mb-binned heatmaps (in 1,000-fold scale), number of ‘non-zero’ interactions (that is, all mappable contacts for satisfying criteria of normal approximation of binomial distribution, see Materials and methods for details), and number of interactions with different frequencies (q-value <0.05) obtained when comparing sperm cells, fibroblasts or ES cell datasets and ‘compressed’ derivations of these datasets. [file 13059_2015_642_MOESM8_ESM.jpeg]

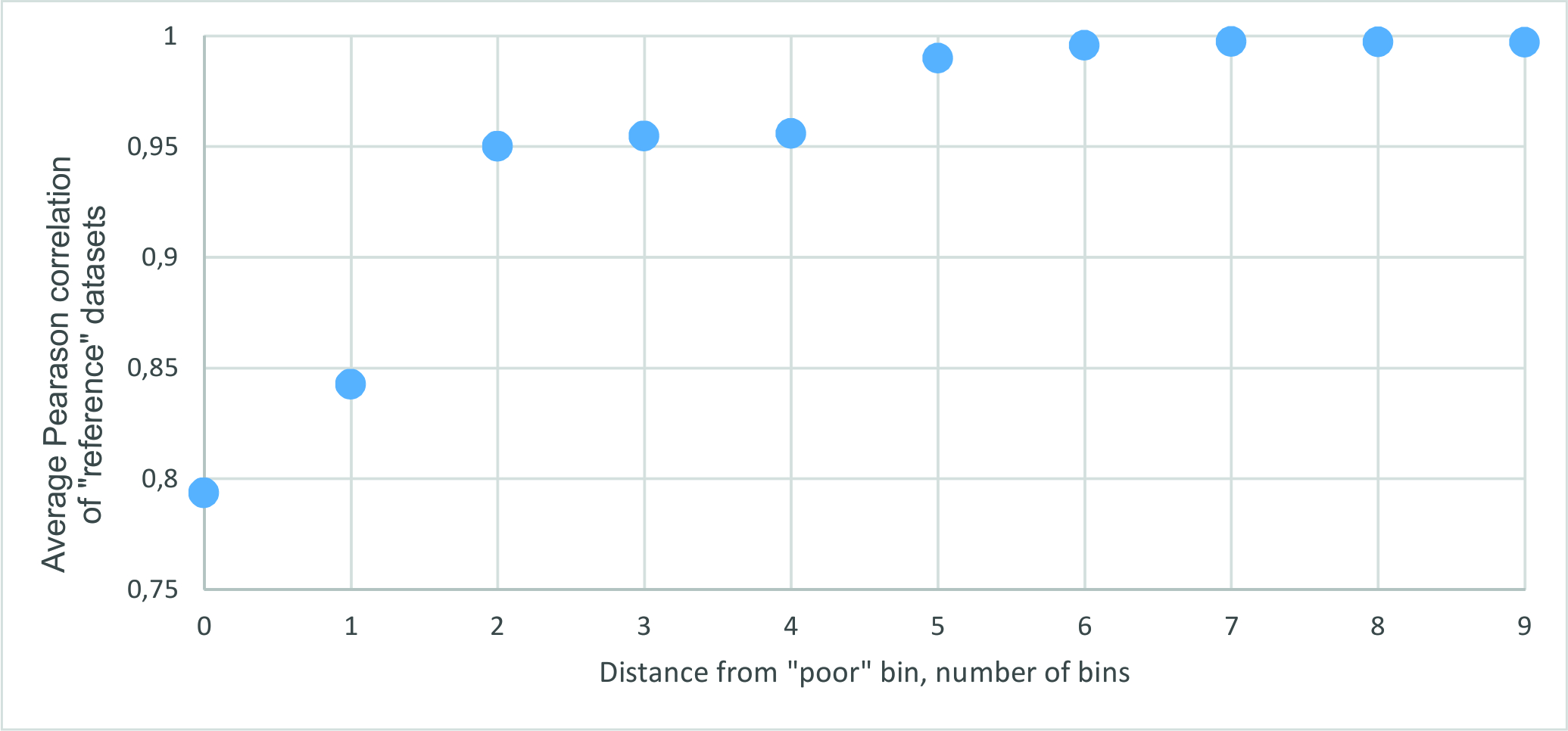

Supplement: Additional file 9: — Nearest to poor bins regions show strong decrease in average Pearson correlation of ‘reference’ datasets. The graph shows average Pearson correlation of ‘reference’ datasets (see ‘Identification of regions different between sperm cells and fibroblasts’ subsection of Materials and methods for definition of ‘reference’ datasets) plotted against distance from poor bins. [file 13059_2015_642_MOESM9_ESM.jpeg]
