# Supplementary material for: Comparison of the three-dimensional organization of sperm and fibroblast genomes using the Hi-C approach
Source: Genome Biol. 2015 Apr 14;16(1):77. doi: 10.1186/s13059-015-0642-0 (PMC4434584; doi:10.1186/s13059-015-0642-0)
Supplement: Additional file 6: — Localization of seven regions on a genome map, as identified by overlapping different datasets of highly dissimilar regions between sperm cells and fibroblasts. [file 13059_2015_642_MOESM6_ESM.docx]

Additional file 6

Localization of seven regions on a genome map, as identified by overlapping different datasets of highly dissimilar regions between sperm cells and fibroblasts

| Chromosome number | Nucleotide start on mouse genome map | Nucleotide end on mouse genome map |
| --- | --- | --- |
| chr5 | 119000000 | 120000000 |
| chr5 | 142000000 | 143000000 |
| chr12 | 35000000 | 36000000 |
| chr13 | 57000000 | 58000000 |
| chr19 | 54000000 | 55000000 |
| chr19 | 8000000 | 9000000 |
| chr19 | 9000000 | 10000000 |
